# Supplementary material for: Universal control of a six-qubit quantum processor in silicon
Source: Nature. 2022 Sep 28;609(7929):919–24. doi: 10.1038/s41586-022-05117-x (PMC9519456; doi:10.1038/s41586-022-05117-x)
Supplement: Supplementary file 1 — Supplementary text, figures, tables, equations and references. [file 41586_2022_5117_MOESM1_ESM.pdf]

nature portfolio

https://doi.org/10.1038/s41586-022-05117-x

Supplementary information

Universal control of a six-qubit quantum processor in silicon

In the format provided by the authors and unedited

SUPPLEMENTARY MATERIALS
Universal control of a six qubit quantum processor in silicon

Stephan G.J. Philips*[1], Mateusz T. Mądzik*[1], Sergey V. Amitonov[1], Sander L. de Snoo[1], Maximilian Russ[1], Nima Kalhor[1], Christian Volk[1], William I.L. Lawrie[1], Delphine Brousse[2], Larysa Tryputen[2], Brian Paquelet Wütz[1], Amir Sammak[2], Menno Veldhorst[1], Giordano Scappucci[1], and Lieven M.K. Vandersypen† [1]

[1] QuTech and the Kavli Institute of Nanoscience, Delft University of Technology, 2600 GA Delft, The Netherlands.

[2] QuTech and Netherlands Organization for Applied Scientific Research (TNO), Delft, The Netherlands.

October 6, 2022

Contents

1 Considerations on scaling **2**
1.1 Scaling limitations of the methods used in the present work . . . 2
1.2 Path forward to larger systems . . . 2

2 Micromagnet design **2**

3 Different samples tested for this experiment **5**

4 Coherence times and visibilities **6**

5 Calibration log **7**

6 Sample design **8**

7 Entanglement Witnesses **9**

8 Quantum state tomography **9**

9 Error channels in the experimental data: dephasing and "heating" **10**
9.1 State tomography simulations . . . 10
9.2 Discussion . . . 11

*These authors contributed equally

†To whom correspondence should be addressed; E-mail: L.M.K.Vandersypen@tudelft.nl

1 Considerations on scaling

1.1 Scaling limitations of the methods used in the present work

We briefly comment on the scaling limitations of some of the methods used in the present experiment, and mention the key ingredients we believe will be necessary for scaling to larger systems.

Micromagnets for EDSR In the present design, there is frequency selectivity along one axis only (its design requirements are discussed in 2). The micromagnet design would need be modified for small 2D arrays in order to get selectively of the qubit frequencies in two dimensions in combination with a sufficiently strong transverse gradient for driving. No micromagnet patterns have been developed that work well in large two-dimensional arrays. Therefore, we believe that a more fruitful path is to use local arrays of a moderate size (where micromagnets can be used and designed to minimize decoherence gradients) and network them together on the same chip using quantum links.

We furthermore note that a known disadvantage of EDSR with micromagnets is that the artificial spin orbit coupling generated by the micromagnets allows charge noise to couple to the spin qubits (this is directly connected to the need for frequency selectivity between neighboring qubits). Also, questions remain on how uniform or reproducible the field of a micromagnet can be across a chip with thousands of micromagnets.

Sensing dots Device A had a sensing dot near the middle of the array in addition to two sensing dots at the end of the array. The sensing dots at the end of the array have the maximum sensitivity to interdot transitions, which we were interested in here. Since we found the full 6-qubit array could be tuned up without the third sensing dot alongside the array, we left it out in devices B and C in order to slightly reduce the complexity of fabrication. Such a third sensor would have made it possible to probe PSB directly on the inner pair of dots. For longer one-dimensional arrays, we would place a sensing dot alongside the qubit array every 3 qubit dots [1].

QND Readout QND can be performed sequentially on multiple qubits, such that multiple qubits can be read out that are too far away from the charge sensors to be read out directly. For instance, in a linear array, one can perform QND readout of qubit 4 by mapping its state onto that of qubit 3, and from there onto that of qubit 2, which is in turn read out using the charge sensor. QND readout of qubit 5 can be done by mapping it onto the state of qubit 4, and so on. This would involve linear overhead in the length of the chain. However, the errors from the successive mapping (CROT/CNOT) operations would accumulate for concatenated QND readout. Alternatively, the number of QND repetitions needed to achieve the target readout fidelity would increase, making the overall overhead a bit higher than linear in the distance. This concept works also in 2D, again with overhead. However, the successive QND measurements would increase the total measurement time, which must remain shorter than the coherence time when implementing quantum error correction schemes.

Alternative readout schemes include CCD like concepts where electrons are shuttled to the sensor one after the other [2] or cascade effects are used [3] to propagate spin-dependent charge motion to the charge sensors.

1.2 Path forward to larger systems

This constitutes a full research topic in itself, which we have spent considerable time thinking about and published several proposal/design papers. For large dense 2D arrays, we have shown that cross-bar addressing techniques can provide universal control and readout [4]. Proposals also exist for bringing individual wires to every qubit in a dense 2D array [5]. Either way, there will still be limits to how large such 2D arrays will work well, for instance because of the limitations of this approach for parallel operations. To scale beyond, we envision networks of local registers [6, 7], connected together with quantum coherent links. Such links can consist of shuttling channels, or perhaps of superconducting resonators, or other methods. An alternative strategy we have explored conceptually is to use highly sparse 2D arrays instead [8]. Also other groups have given the operation of such on-chip spin qubit networks detailed consideration, see e.g. [9].

2 Micromagnet design

Most micromagnets used in spin qubit experiments have been designed to support up to 2 to 3 qubits [10, 11, 12]. In this experiment, a suitable gradient was needed across a six-qubit device. A schematic of the micromagnet design, along with the coordinate system is shown in Supplementary Fig. 1. When designing the magnet, we set the following requirements:

- Target Rabi frequency of 5-10 MHz. The higher the driving speed, the more operations can be completed within the coherence time. We limit the target frequency to 10 MHz as we have found the Rabi frequency to become non-linear

in the driving amplitude for higher Rabi frequencies (see also ref. [13]). Based on prior experience, this translates to a target gradient in the transverse component of the micromagnet stray field of at least 1 mT/nm.

- All the qubits are driven by a single gate using frequency multiplexing. Overlap in resonance frequencies must thus be avoided. We target a frequency difference of 100 MHz between different qubits when the micromagnets are fully magnetized. This value is chosen as it results in an acceptable level of crosstalk when driving the qubits at a Rabi frequency of 5-10 MHz.

- Following [13], we aim at a maximal decoherence gradient ($|\frac{\partial B_z}{\partial z}| + |\frac{\partial B_z}{\partial y}|$) of 0.1 mT/nm. In this way, qubit coherence is not impacted assuming an intrinsic qubit linewidth of $\delta f \sim 10$ kHz. We report the decoherence gradient calculated at the center of the quantum dot location.

To design the micromagnets, we parametrized a model of the magnets and optimized a number of design parameters (e.g. the magnet-magnet separation, height of the magnets, size and angle of the slanting part and distance from the quantum well). The core of the calculations was based on the python package magpylib [14], which allows for fast analytical solutions of simple magnet geometries (our wrapper can be found at [15]). We assume full magnetization of the micromagnet in our simulations. The result of the optimization is shown in Supplementary Fig. 1 b-f. In panel b-c, one can visually inspect the micromagnet stray magnetic field profile. Panel d-f show line cuts of magnetic properties described above, along the length of the six-qubit array. We are able to satisfy all our design targets.
Supplementary Fig. 1g-i show the measured frequency distributions of the qubits on three samples from different fabrication runs. The measured frequency profile for sample A is comparable to the simulated profile of Supplementary Fig. 1d. For samples B and C, the measured frequencies are very different than expected based on the simulations. The parabolic trend seen in Sample C (the sample discussed in the main text paper) can be roughly reproduced by assuming that the magnet boundary is displaced. The solid white lines in Supplementary Fig. 1j show the bottom and the top of the magnet, indicating that the micromagnet sidewalls are slightly tilted, which indeed effectively displaces the magnet boundary. When simulating the frequency profile with the black dashed line as the magnet boundary (as a rough approximation), we obtain the profile shown in purple in Supplementary Fig. 1i. The qubit frequencies reported in these plots are reproducible between different cooldowns of the same device.

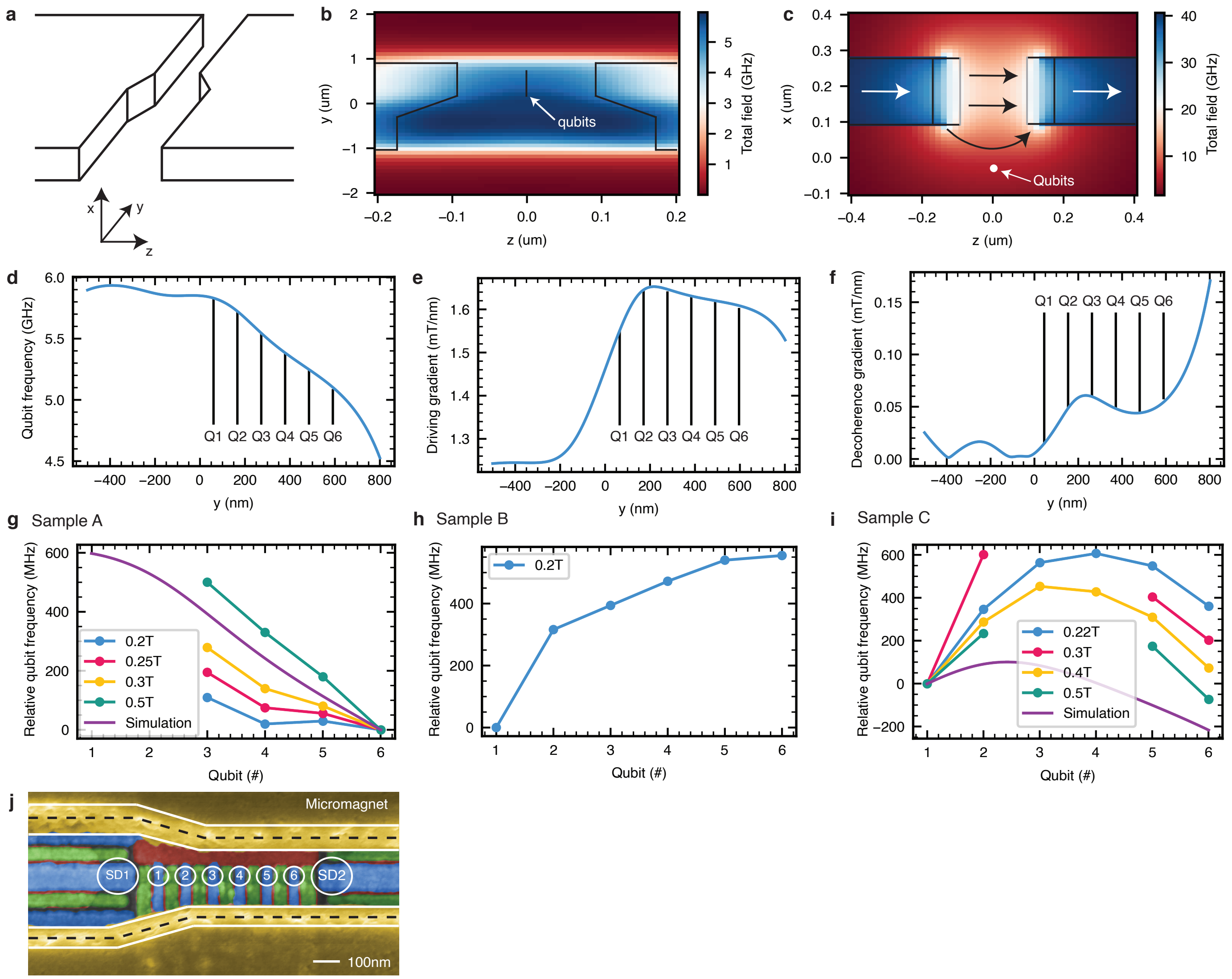

Supplementary Figure 1 | Micromagnet simulations and experimental values a, Sketch of the micromagnet structure in 3D. The micromagnets are magnetized along the z direction, with a magnetization vector of M = (0 T, 0 T, 1.5 T) as estimated from the experiments. The maximum magnetisation is reached in the experiment when 0.1-0.2T external field is applied to the magnets. **b**, Simulation of the total field generated by the micromagnets in the z,y plane, where the quantum dots reside. A projection of the micromagnets is shown by the black lines overlaid in the figure. **c**, Simulation of the total field generated by the micromagnets in the z,x plane. **d**, Simulated values for the qubit frequencies (without the contribution of the externally applied magnetic field) along the y axis, with the target qubit positions indicated. **e**, Simulated values for the (transverse) driving gradient of the micromagnet ($|\frac{\partial B_x}{\partial z}| + |\frac{\partial B_y}{\partial z}|$) along the y axis, with the target qubit positions indicated. **f**, Simulated values of the decoherence gradient of the micromagnet ($|\frac{\partial B_z}{\partial z}| + |\frac{\partial B_z}{\partial y}|$) along the y axis, with the target qubit positions indicated. **g-i** Experimentally measured qubit frequencies for three different samples with the same micromagnet design. All frequencies in the plots are taken in reference to one qubit, to clearly display the difference in qubit frequency distribution versus the different fields applied to the sample. The simulated curve in panel **i** is computed assuming the magnet boundary is as indicated by the dashed black line in panel **j**. **j**, False colored SEM image of a sample similar to sample C and fabricated in the same fabrication run. The white lines indicates the top and bottom edge of the micromagnet, with inner lines indicating the intended step boundary. The black, dashed line is the average distance between top and bottom edge of the magnets.

3 Different samples tested for this experiment

	Device A	Device B	Device C
	**Design**		
Number of qubits	6	6	6
Number of sensing dots	3	2	2
Access to reservoir from dots	1, 6	1, 3, 4, 6	1, 6
Dot pitch	100 nm	80 nm	90 nm
Tuning for sufficient t_c	3,1,3,1,3,1	1,1,1,1,1,1	1,1,1,1,1,1
Comment	Right SD unusable due to faulty source contact		
	**Valley splitting** (μeV)		
Substrate [16]	Quantum well A	Quantum well B	Quantum well B
Dot 1	160	118.9	220
Dot 2	130	160.8	140
Dot 3	0	113.4	105
Dot 4	173	160.9	138
Dot 5	–	56.8	220
Dot 6	–	148.4	300
Comment	Valley splitting in dot 3 too low for qubit experiments		
	**Dephasing time T_2^*** (μs)		
Dot 1	12.7	3.0	3.0
Dot 2	5.5	3.2	2.5
Dot 3	3.4	6.0	3.7
Dot 4	7.2	3.0	3.7
Dot 5	–	6.5	5.9
Dot 6	–	6.4	5.1
	**Hahn echo decay time T_2^{h}** (μs)		
Dot 1	24.8	77	14.0
Dot 2	28.1	47	21.1
Dot 3	–	75	40.1
Dot 4	26.9	48	37.2
Dot 5	–	58	44.7
Dot 6	–	41	26.7
	**General comment**		
	Sample discarded due to low valley splitting in dot 3	Sample discarded due to low EDSR drive quality	Sample used in this experiment

Supplementary Data Table 1 | Properties of different samples tested for this experiment. In the course of this experiment, we have modified the sample design to match the requirements set for 6-qubit control. Device A has been discarded due to the low valley splitting in dot 3. Additionally, we could only use 4 out of 6 quantum dots, due to a failure on the right sensing dot. Due to the 100 nm dot pitch, it was necessary to tune the device to the (3,1,3,1,3,1) charge configuration in order to achieve sufficient tunnel coupling t_c between adjacent dots. Device B allowed for 6-qubit operation, however, the EDSR drive quality was poor, with Rabi oscillations decaying within a few periods. We have used Device C with a 90 nm pitch for the data presented in the main text. The reported valley splittings are measured using magnetospectroscopy. Since there are no reservoirs available in the middle of the device, we measure the valley splitting by performing spectroscopy on the anticrossing between the (2,0) and (1,1) at a relatively low tunnel coupling.

4 Coherence times and visibilities

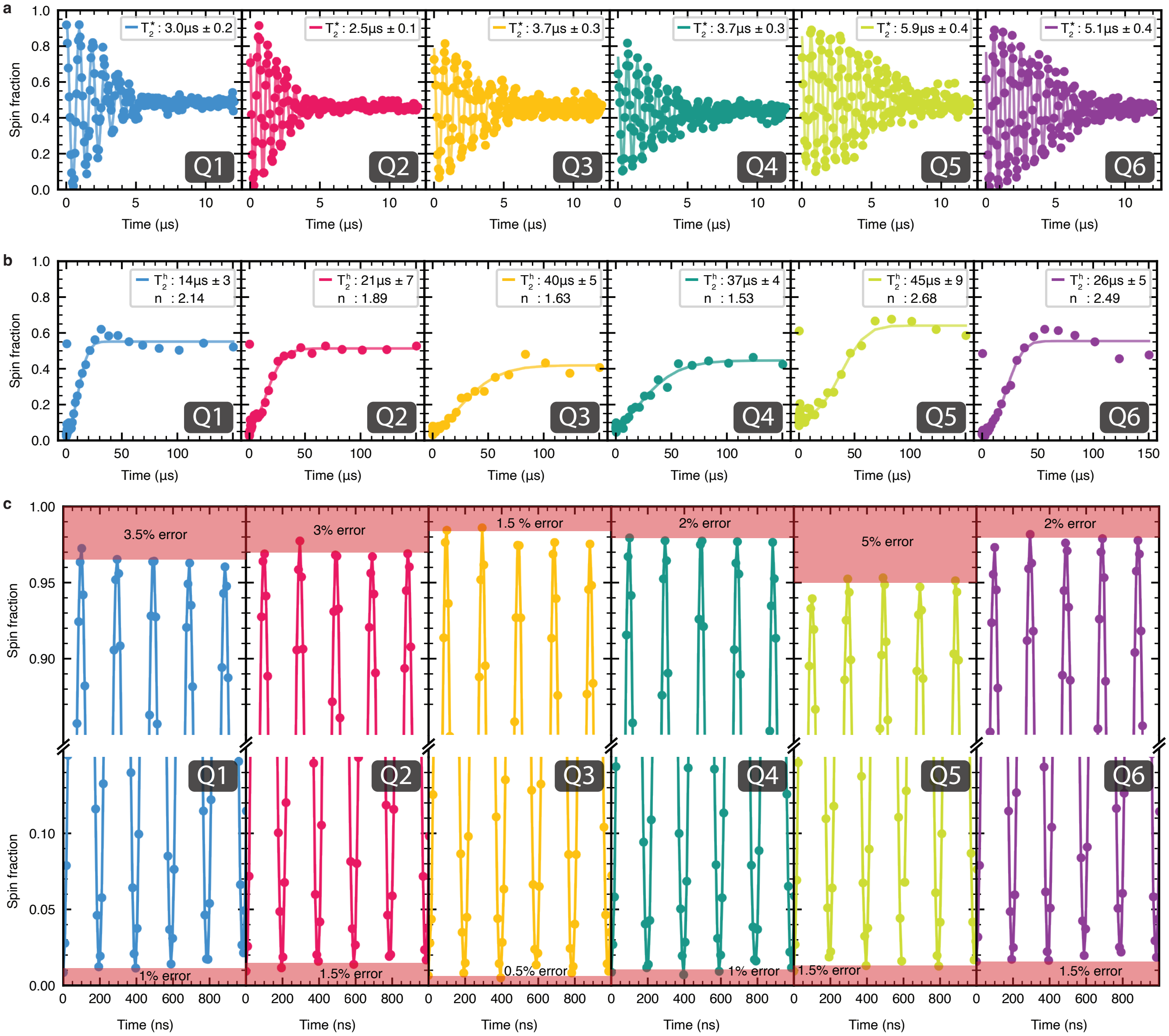

Supplementary Figure 2 | $\mathbf{T_2^*}$, $\mathbf{T_2^h}$ and visibility measurements a, T_2^* measurement for each qubit. These measurements were measured without any pre-pulse and are fitted to a Gaussian decay: $P_s(t) = A\cos(\omega t + \phi)\exp\left(-\frac{t^2}{T_2^{*2}}\right) + B$. **b** T_2^h measurement for each qubit. These measurements are also performed without any pre-pulse and are fitted using : $P_s(t) = A\exp\left(-\frac{t^n}{T_2^{h\ n}}\right) + B$. **c**, Detailed plots of the Rabi oscillations shown in the main text. The errors contributing to loss of visibility are estimated by eye.

5 Calibration log

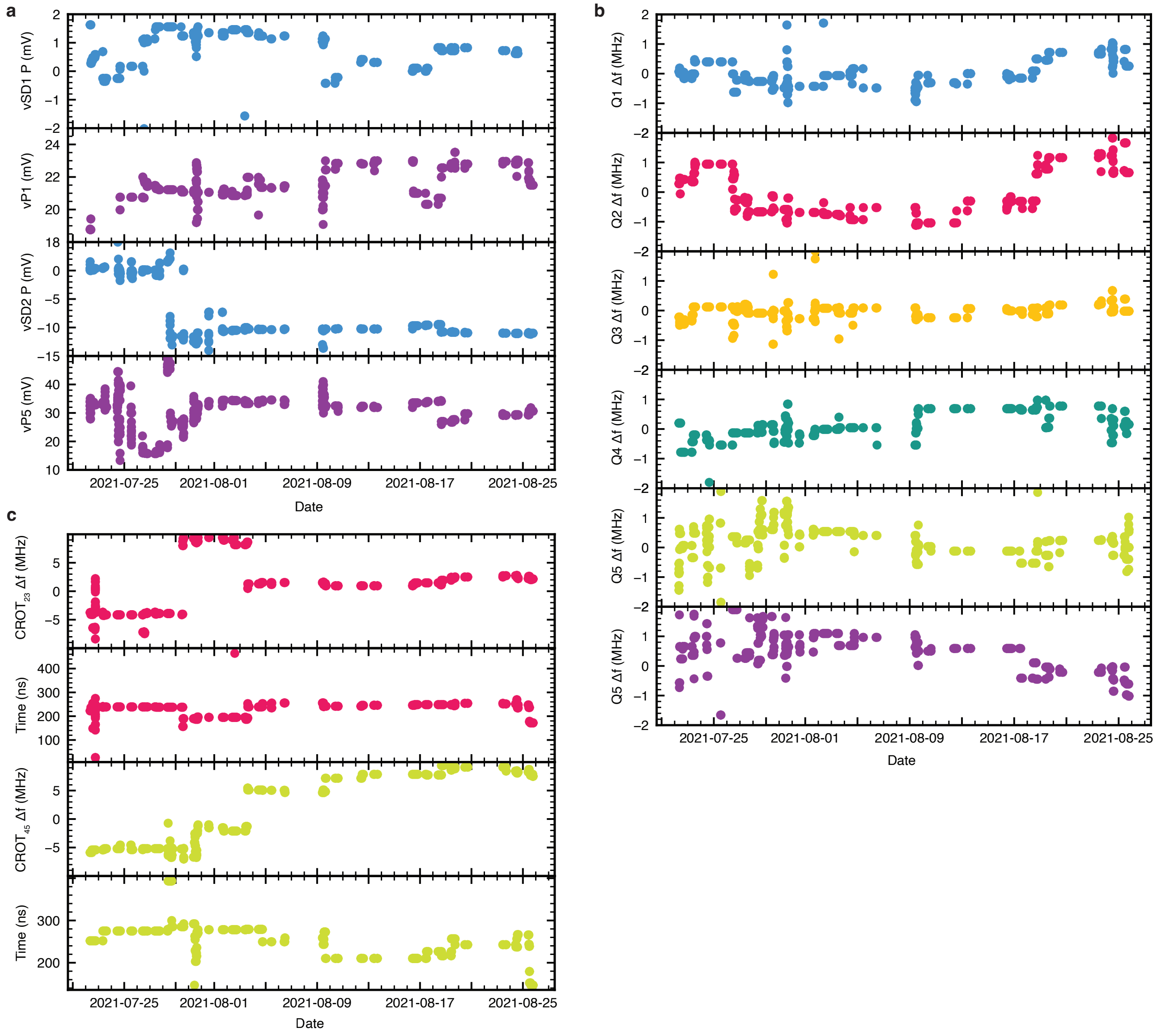

Supplementary Figure 3 | Calibration log of the sample during a period of approximately one month. Every data point in the calibration plot represents a result of a calibration measurement **a**, Calibration results of the sensing dot and readout point. The panels with the blue dots show the calibration results of the optimal operating point of SD1 (top) and SD2 (bottom). The purple dots show the readout point used for the parity readout for dot pair 12 (top) and 56 (bottom). **b**, Calibration results for the qubit resonance frequency, one plot per qubit. We plotted the deviation from the average resonance frequency. **c**, Controlled rotation calibration. The calibrated values for the driving frequency and burst duration for $CROT_{23}$ and $CROT_{45}$ are shown.

6 Sample design

A CAD image of the device design in shown in Supplementary Fig. 4a,b. The samples are designed without a physical mesa. A mesa is often used to prevent leakage at the bondpads where damage by a wirebonder could create a contact to the 2DEG residing below. Instead, we deposit a 200 nm silicon nitride layer underneath the bondpads to prevent leakage to the 2DEG below (Supplementary Fig. 4c). In addition, all the gates in the plunger and barrier gate layer run over a screening gate layer, blocking any current flow between the bondpad and the center of the device, as long as the screening gates are not accumulated (Supplementary Fig. 4d). As a last measure, part of the gate fan-out wiring is reduced to patterned nanowires (Supplementary Fig. 4e), which need higher voltages to accumulate.

We designed an on-chip coplanar waveguide with a characteristic impedance of 50 Ohm to optimize the power delivery of the microwave excitation. For the RF readout, we use a low capacitance design where the readout signal is applied via the source contact [17, 18, 19]. We ensure low parasitic capacitance by placing the source contact close to the center of the device ((Supplementary Fig. 4a)) and wire bond directly to these source contacts. Furthermore, running the accumulation gates over a screening gate prevents the creation of a large capacitance between accumulated 2DEG and the accumulation gate, which helps achieve a good RF readout.

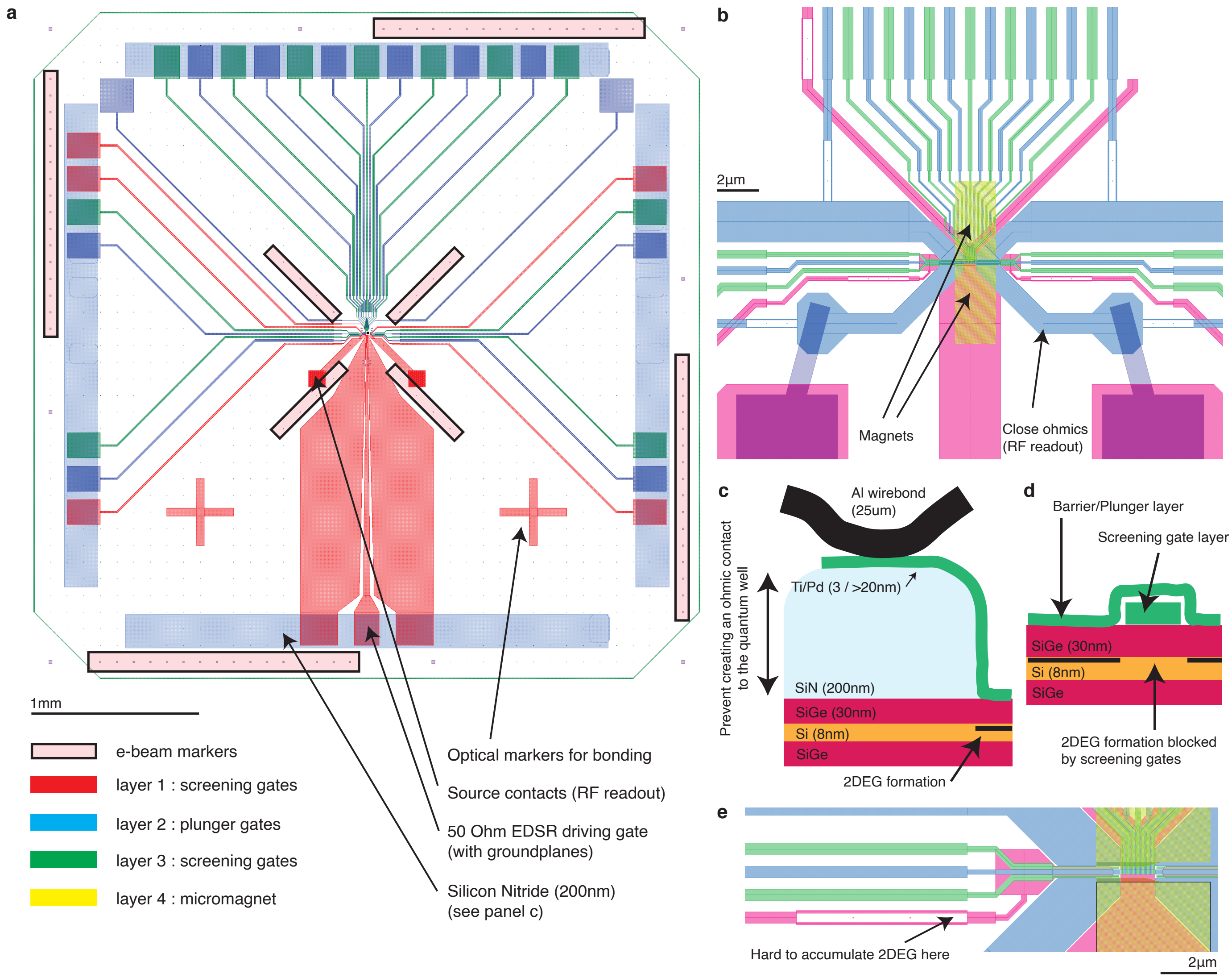

Supplementary Figure 4 | Sample design a, CAD image of the sample layout. **b**, Zoom in of **a**, showing the active region of the device. **c**, Silicon nitride (SiN) below the bondpad prevents punch-through of the bondwire to the quantum well. **d**, Screening gates block current flow below the gates between the bondpads and the active device area. **e**, Narrow segments of the gates increase the accumulation voltage underneath, and thereby also block current flow underneath.

7 Entanglement Witnesses

Entanglement witnesses are an efficient means to check whether a state is entangled. A witness is constructed in such a manner that its expectation value is negative in case the state is entangled. When choosing an entanglement witness, usually one needs to balance the number of operators measured versus the possible states that can be detected. Examples for GHZ states include the optimal, stabilizer and Mermin witness [20]. We choose a witness which requires a few more measurements but is able to detect entanglement across a larger part of the space with entangled states:

$$W = III - |\psi_{\text{GHZ}}\rangle \langle\psi_{\text{GHZ}}| \ . \tag{1}$$

This operator can be decomposed into Pauli operators. For three qubits, this results in:

$$\begin{aligned} W =& \frac{3}{8}\langle III\rangle - \frac{1}{8}\langle IZZ\rangle - \frac{1}{8}\langle XXX\rangle + \frac{1}{8}\langle XYY\rangle + \\ & \frac{1}{8}\langle YXY\rangle + \frac{1}{8}\langle YYX\rangle - \frac{1}{8}\langle ZIZ\rangle - \frac{1}{8}\langle ZZI\rangle \ . \end{aligned} \tag{2}$$

We use similar procedures as for the state tomography to remove the SPAM error of the witness operators.

8 Quantum state tomography

Quantum state tomography is used to obtain the density matrices for the qubits. We used the maximum likelihood method to obtain the density matrices reported in this paper.

The concept of quantum state tomography is based on the idea that every density matrix can be decomposed in a set of orthogonal basis states (e.g. Pauli basis):

$$\rho = |\psi\rangle\langle\psi| = \frac{1}{2^N}\sum_i^{4^N} \langle\psi|\hat{V}_i|\psi\rangle \cdot \hat{V}_i \ , \tag{3}$$

where $\hat{V}_i$ is the ith basis state. Here $\hat{V}_i$ are of the form $\{I, X, Y, Z\}^{\otimes N}$, and N is the number of qubits. In the experiment we measure the expectation value M_i of all the possible $\hat{V}_i$'s; $M_i = \langle\psi| V_i |\psi\rangle$ and reconstruct a first estimate of the density matrix using equation 3. To ensure that the obtained density matrix ρ is valid, we take the closest positive semi-definite matrix and ensure that the norm of ρ is one.

The resulting state can be used as input for the Maximum Likelihood Estimation (MLE) [21]. This is procedure to obtain the most likely state for a given set of M_i, using an optimizer for the following cost function:

$$\sum_i^{4^N} |M_i - tr(\rho V_i)| \tag{4}$$

To make this method work well, we use so called T matrices, which is the lower triangular matrix, where every non-zero entry is a variable. The relation to the density matrix is $\rho = TT^\dagger$. An initial guess of T is obtained by performing a Cholesky decomposition on the density matrix used as input for MLE.

The fidelity of the estimated density matrix can be further increased by taking into account the readout errors of the system. When measuring an observable, the following matrix can be used to describe the measurement process:

$$P_{\text{meas}} = S_k P_{\text{real}} = \begin{pmatrix} F_{k,-1} & 1 - F_{k,+1} \\ 1 - F_{k,-1} & F_{k,+1} \end{pmatrix} P_{\text{real}} \tag{5}$$

where P_{meas} are the measured spin spin probabilities for the kth observable (while we are interested in the real probability amplitudes). $P_{\text{real}}\dot{F}_{k,-1}$ ($F_{k,+1}$) is the corrected for readout error probability to obtain -1 ($+1$) as measurement outcome for the kth observable. These numbers are derived from the visibility of single-qubit Rabi oscillations such as in main text Fig. 2, but taken with the same initialization and readout sequences as are used in the circuits for preparing the density matrix.

The following observables are measured:

k	observable
1	$ZIIIII$
2	$IZIIII$
3	$ZZIIII$
4	$IIZIII$
5	$IIIZII$
6	$IIIIZI$
7	$IIIIIZ$
8	$IIIIZZ$

and have their own associated S_k matrix. We decompose other measurement operators we want to measure into elements of this set, for example $ZZZIII$ is decomposed into a combination of the $ZZIIII$ and $IIZIII$ operators, which we can simultaneously measure. The procedure we would use to generate to expectation value would be give by :

$$\langle ZZZIII \rangle = \mathrm{Tr}(ZZ \cdot (S_3 \otimes S_4)^{-1} \cdot P_{\mathrm{meas}}) \tag{6}$$

Where P_{meas} is a vector with the probabilities of the four possible measurement outcomes.

A comment needs to be made on the validity of this approach, as the SPAM errors are most likely spread over initialization and readout. The above method of removing readout errors could then artificially result in faulty results (e.g. expectation values above 1) in specific circumstances. As an example, let us assume that initialization and readout are error-prone for one qubit (Q1) and perfect for the other qubit (Q2). Characterization of SPAM errors would give us a matrix S_1 with which to correct the measurement outcomes for Q1.

Now consider performing state tomography after running a CNOT or SWAP operation:

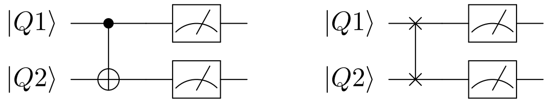

In the first case, correction using the matrix S_1 will accurately remove the SPAM errors from the measured values for Q1. For qubit two, errors have been propagated from Q1, but these errors are not removed by the analysis as there are no SPAM errors on Q2 by itself. In this case, SPAM error removal thus only removes a subset of the errors introduced by SPAM. In the second case, we get for both qubits the wrong results. For Q1 the corrected expectation values could exceed one (which would be non-physical), whereas for Q2 there is no correction even though initialization errors on Q1 have propagated to Q2. In the experiments that are performed in this work, the circuits focus on entangling rather than swapping states. For that reason we believe that we do not introduce nonphysical elements by applying the SPAM correction; rather it cannot remove all errors and will tend to worsen the actual fidelity.

9 Error channels in the experimental data: dephasing and "heating"

In order to examine the impact of dephasing on the measured density matrices of main text Figs. 4-5, we compare with the results of two numerical simulations performed according to the following methodology.

9.1 State tomography simulations

The multi-qubit system is well approximated by the Hamiltonian

$$H = H_{\mathrm{Zeeman}} + H_{\mathrm{Heisenberg}}, \tag{7}$$

consisting of the Zeeman interaction

$$H_{\mathrm{Zeeman}} = \sum_{j=1}^{6} \frac{g_j \mu_B}{2} \boldsymbol{B}_j \cdot \boldsymbol{\sigma}_j \tag{8}$$

and the isotropic Heisenberg exchange interaction

$$H_{\mathrm{Heisenberg}} = \sum_{\langle j,k \rangle} \frac{J_{j,k}}{4} \boldsymbol{\sigma}_j \cdot \boldsymbol{\sigma}_k. \tag{9}$$

Here, $\boldsymbol{\sigma}_j$ is the vector of the Pauli matrices acting on qubit j, $\boldsymbol{B}_j = (v_{ac,j}(t)\partial B_{x,j}\cos(2\pi f_j), 0, B_{z,j})^T$ is the combined magnetic field, $\partial B_{x,j}$ the micromagnet gradient orthogonal to the qubit array axis (expressed in units of Tesla/V), $v_{ac,j}$ the voltage amplitude applied for driving EDSR, and $J_{j,k} = J_{\text{res},j,k}\exp(2\alpha_{j,k}v_{B,j,k}(t))$ is the exchange interaction between qubit j and k, where $J_{\text{res},j,k}$ is the residual exchange for $v_{B,j,k}(t)) = 0$. The sum $\langle j,k\rangle$ runs over all neighboring pairs in the linear array.

For the numerical simulations, we solve the time-dependent Schrödinger equation

$$i\hbar\frac{d}{dt}|\psi(t)\rangle = H|\psi(t)\rangle \tag{10}$$

by discretizing $H(t+\Delta t)$ into segments of length Δt taking $H(t)$ constant in the time-interval $[t, t+\Delta t]$. We compute the unitary propagator according to

$$U(t+\Delta t) = e^{-\frac{i}{\hbar}H(t+\Delta t)}U(t), \tag{11}$$

where $\hbar = h/(2\pi)$ is the reduced Planck constant. The simulations are performed in the multiply rotating frame which removes the Larmor precession of each qubit around the average external magnetic field $\sum_{j=1}^{N} B_{z,j}/N$. By making the so-called rotating wave approximation (RWA) we neglect counter-rotating terms such that we can choose $\Delta t = 10$ ps as a sufficiently small time step.

The simulated GHZ states are the final states $\rho_f = U_f\rho_{\text{init}}U_f^\dagger$ obtained by applying the pulse sequence of main text Fig. 5a to the ideal input state ρ_{init} (here U_f is shorthand for the product of the successively applied unitary operations). For simplicity each GHZ simulation is performed on the subspace of only the involved qubits, motivated by the small residual exchange to the other qubits.

Low-frequency noise is included in the simulation via quasistatic fluctuations $H_{\text{Zeeman}} \to H_{\text{Zeeman}} + \sum \xi_J\sigma_{z,j}$ and averaging the final result over 5000 random initializations. Here ξ_J are Gaussian stochastic variables with mean $\langle\xi_J\rangle = 0$ and variance $\langle\xi_J^2\rangle = h^2/2(\pi \mathrm{T}_{2,j}^*)^2$.

9.2 Discussion

In the tables below we present a comparison of the experimental results (RAW data: without SPAM error removal; Processed data: with SPAM error removal) to the outcomes of the two simulations with different input parameters. The first simulation uses the T_2^* values from the table in main text Fig. 2e. The second simulation takes the T_2^* values obtained after a 4 µs microwave bursts applied off-resonance before the pulse sequence used to measure T_2^*. We find systematically that such a prepulse causes both a shift in the qubit frequency and a reduction in T_2^*, by amounts that depend on the microwave burst duration and power (see Extended Data Fig. 7). In the actual experiments, prepulses shorter than 4 µs are used, to strike a balance between saturating the frequency shift and not reducing T_2^* too much. We can thus expect that dephasing reduces the off-diagonal elements in the measured density matrices of Fig. 4-5 by an amount that lies in between the case of the two simulations, with the shorter and longer sets of T_2^* values. Furthermore, the longer the state preparation sequence, the shorter the resulting T_2^*, which is consistent with the fact that the off-diagonal entries are lower for the GHZ states of qubits 2-4 and 3-5 than for GHZ states of qubits 1-3 or 4-6.

The diagonal entries deviate most from the ideal expectation for the GHZ states involving qubits 2-4 and 3-5. State preparation of qubits 1-3 or qubits 4-6 by itself works very well, but we find consistently that state preparation of qubits 4-6 is somewhat degraded when it follows state preparation of qubits 1-3 (see Extended Data Fig. 3), and vice versa. In the simulations we assume perfect initial states in order to probe only the effects of dephasing.

Finally, the qubit frequency shifts from the prepulses and the pulses applied during state preparation lead to reproducible phase shifts seen in the experimentally prepared GHZ states, deviating from the ideal GHZ state $(|000\rangle + |111\rangle)/\sqrt{2}$.

The frequency shifts and reduced dephasing times generally bear signatures of heating, as in previous reports [22, 23]. Their microscopic origin is at present not well understood.

Generally, we find that the reduced coherence times in the second simulation provides us with state fidelities closely resembling the experimental data after SPAM removal. We neglect the non-ideal phase component in the experimental data when making this comparison, as we have omitted frequency shits and virtual-Z gates in our simulation.

Qubits 12

	Density Matrices	Fidelity
Raw data	$\begin{pmatrix} 0.46e^{0.0i} & 0.047e^{-1.989i} & 0.063e^{1.827i} & 0.414e^{-0.0i} \\ 0.047e^{1.989i} & 0.034e^{0.0i} & 0.02e^{-0.016i} & 0.018e^{-2.087i} \\ 0.063e^{-1.827i} & 0.02e^{0.016i} & 0.03e^{0.0i} & 0.104e^{-1.708i} \\ 0.414e^{0.0i} & 0.018e^{2.087i} & 0.104e^{1.708i} & 0.476e^{0.0i} \end{pmatrix}$	88.2%
Processed data	$\begin{pmatrix} 0.461e^{0.0i} & 0.046e^{-2.008i} & 0.07e^{1.814i} & 0.424e^{0.001i} \\ 0.046e^{2.008i} & 0.03e^{0.0i} & 0.016e^{-0.052i} & 0.013e^{-2.489i} \\ 0.07e^{-1.814i} & 0.016e^{0.052i} & 0.03e^{0.0i} & 0.106e^{-1.721i} \\ 0.424e^{-0.001i} & 0.013e^{2.489i} & 0.106e^{1.721i} & 0.479e^{0.0i} \end{pmatrix}$	89.4%
Full T_2^* simulation	$\begin{pmatrix} 0.502e^{0.0i} & 0.004e^{1.455i} & 0.017e^{-2.195i} & 0.468e^{-0.096i} \\ 0.004e^{-1.455i} & 0.004e^{-0.0i} & 0.003e^{0.095i} & 0.005e^{-1.923i} \\ 0.017e^{2.195i} & 0.003e^{-0.095i} & 0.004e^{0.0i} & 0.017e^{2.172i} \\ 0.468e^{0.096i} & 0.005e^{1.923i} & 0.017e^{-2.172i} & 0.49e^{-0.0i} \end{pmatrix}$	96.2%
Reduced T_2^* simulation	$\begin{pmatrix} 0.499e^{0.0i} & 0.003e^{1.687i} & 0.017e^{-2.074i} & 0.405e^{-0.093i} \\ 0.003e^{-1.687i} & 0.009e^{0.0i} & 0.007e^{0.073i} & 0.005e^{-2.411i} \\ 0.017e^{2.074i} & 0.007e^{-0.073i} & 0.01e^{0.0i} & 0.015e^{2.118i} \\ 0.405e^{0.093i} & 0.005e^{2.411i} & 0.015e^{-2.118i} & 0.483e^{0.0i} \end{pmatrix}$	89.4%

Supplementary Data Table 2 | Experimental vs simulated state tomography for qubits 12

Qubits 23

	Density Matrices	Fidelity
Raw data	$\begin{pmatrix} 0.105e^{0.0i} & 0.057e^{0.812i} & 0.054e^{0.611i} & 0.029e^{-0.82i} \\ 0.057e^{-0.812i} & 0.425e^{0.0i} & 0.403e^{0.091i} & 0.052e^{0.74i} \\ 0.054e^{-0.611i} & 0.403e^{-0.091i} & 0.449e^{0.0i} & 0.066e^{0.659i} \\ 0.029e^{0.82i} & 0.052e^{-0.74i} & 0.066e^{-0.659i} & 0.021e^{0.0i} \end{pmatrix}$	83.8%
Processed data	$\begin{pmatrix} 0.075e^{0.0i} & 0.066e^{0.796i} & 0.058e^{0.614i} & 0.02e^{-0.658i} \\ 0.066e^{-0.796i} & 0.439e^{0.0i} & 0.451e^{0.091i} & 0.053e^{0.754i} \\ 0.058e^{-0.614i} & 0.451e^{-0.091i} & 0.471e^{0.0i} & 0.062e^{0.633i} \\ 0.02e^{0.658i} & 0.053e^{-0.754i} & 0.062e^{-0.633i} & 0.016e^{0.0i} \end{pmatrix}$	90.4%
Full T_2^* simulation	$\begin{pmatrix} 0.01e^{0.0i} & 0.032e^{-1.457i} & 0.032e^{-1.573i} & 0.009e^{-0.095i} \\ 0.032e^{1.457i} & 0.481e^{0.0i} & 0.471e^{0.044i} & 0.017e^{1.246i} \\ 0.032e^{1.573i} & 0.471e^{-0.044i} & 0.5e^{0.0i} & 0.015e^{1.513i} \\ 0.009e^{0.095i} & 0.017e^{-1.246i} & 0.015e^{-1.513i} & 0.008e^{0.0i} \end{pmatrix}$	96.1%
Reduced T_2^* simulation	$\begin{pmatrix} 0.029e^{0.0i} & 0.027e^{-1.37i} & 0.03e^{-1.944i} & 0.024e^{-0.089i} \\ 0.027e^{1.37i} & 0.463e^{0.0i} & 0.416e^{0.054i} & 0.022e^{0.639i} \\ 0.03e^{1.944i} & 0.416e^{-0.054i} & 0.482e^{0.0i} & 0.012e^{1.514i} \\ 0.024e^{0.089i} & 0.022e^{-0.639i} & 0.012e^{-1.514i} & 0.026e^{0.0i} \end{pmatrix}$	88.8%

Supplementary Data Table 3 | Experimental vs simulated state tomography for qubits 23

Qubits 34

	Density Matrices	Fidelity
Raw data	$\begin{pmatrix} 0.149e^{0.0i} & 0.04e^{1.2i} & 0.055e^{-0.241i} & 0.025e^{1.213i} \\ 0.04e^{-1.2i} & 0.395e^{0.0i} & 0.377e^{-0.173i} & 0.104e^{-1.446i} \\ 0.055e^{0.241i} & 0.377e^{0.173i} & 0.424e^{0.0i} & 0.092e^{-1.393i} \\ 0.025e^{-1.213i} & 0.104e^{1.446i} & 0.092e^{1.393i} & 0.032e^{0.0i} \end{pmatrix}$	78.0%
Processed data	$\begin{pmatrix} 0.072e^{0.0i} & 0.048e^{0.805i} & 0.055e^{0.08i} & 0.009e^{0.598i} \\ 0.048e^{-0.805i} & 0.434e^{0.0i} & 0.444e^{-0.182i} & 0.105e^{-1.531i} \\ 0.055e^{-0.08i} & 0.444e^{0.182i} & 0.466e^{0.0i} & 0.103e^{-1.389i} \\ 0.009e^{-0.598i} & 0.105e^{1.531i} & 0.103e^{1.389i} & 0.028e^{0.0i} \end{pmatrix}$	88.6%
Full T_2^* simulation	$\begin{pmatrix} 0.004e^{0.0i} & 0.017e^{-0.771i} & 0.017e^{-1.079i} & 0.003e^{0.014i} \\ 0.017e^{0.771i} & 0.49e^{0.0i} & 0.485e^{-0.264i} & 0.003e^{0.832i} \\ 0.017e^{1.079i} & 0.485e^{0.264i} & 0.503e^{0.0i} & 0.003e^{1.344i} \\ 0.003e^{-0.014i} & 0.003e^{-0.832i} & 0.003e^{-1.344i} & 0.003e^{0.0i} \end{pmatrix}$	96.5%
Reduced T_2^* simulation	$\begin{pmatrix} 0.009e^{-0.0i} & 0.014e^{-0.723i} & 0.015e^{-1.134i} & 0.007e^{-0.002i} \\ 0.014e^{0.723i} & 0.485e^{0.0i} & 0.429e^{-0.261i} & 0.004e^{0.255i} \\ 0.015e^{1.134i} & 0.429e^{0.261i} & 0.499e^{0.0i} & 0.002e^{0.823i} \\ 0.007e^{0.002i} & 0.004e^{-0.256i} & 0.002e^{-0.827i} & 0.008e^{-0.0i} \end{pmatrix}$	90.6%

Supplementary Data Table 4 | Experimental vs simulated state tomography for qubits 34

Qubits 45

	Density Matrices	Fidelity
Raw data	$\begin{pmatrix} 0.049e^{0.0i} & 0.026e^{-3.07i} & 0.035e^{-2.819i} & 0.012e^{-1.345i} \\ 0.026e^{3.07i} & 0.469e^{0.0i} & 0.444e^{-0.125i} & 0.033e^{-0.174i} \\ 0.035e^{2.819i} & 0.444e^{0.125i} & 0.476e^{0.0i} & 0.037e^{0.005i} \\ 0.012e^{1.345i} & 0.033e^{0.174i} & 0.037e^{-0.005i} & 0.006e^{0.0i} \end{pmatrix}$	91.3%
Processed data	$\begin{pmatrix} 0.025e^{0.0i} & 0.028e^{-3.057i} & 0.037e^{-2.829i} & 0.006e^{-1.595i} \\ 0.028e^{3.057i} & 0.481e^{0.0i} & 0.481e^{-0.125i} & 0.036e^{-0.181i} \\ 0.037e^{2.829i} & 0.481e^{0.125i} & 0.489e^{0.0i} & 0.038e^{0.027i} \\ 0.006e^{1.595i} & 0.036e^{0.181i} & 0.038e^{-0.027i} & 0.005e^{0.0i} \end{pmatrix}$	96.2%
Full T_2^* simulation	$\begin{pmatrix} 0.002e^{0.0i} & 0.008e^{0.103i} & 0.007e^{0.12i} & 0.002e^{-0.152i} \\ 0.008e^{-0.103i} & 0.491e^{0.0i} & 0.493e^{-0.005i} & 0.015e^{-1.539i} \\ 0.007e^{-0.12i} & 0.493e^{0.005i} & 0.505e^{0.0i} & 0.016e^{-1.596i} \\ 0.002e^{0.152i} & 0.015e^{1.539i} & 0.016e^{1.596i} & 0.002e^{0.0i} \end{pmatrix}$	99.2%
Reduced T_2^* simulation	$\begin{pmatrix} 0.009e^{0.0i} & 0.009e^{0.122i} & 0.004e^{0.402i} & 0.009e^{-0.032i} \\ 0.009e^{-0.122i} & 0.483e^{0.0i} & 0.472e^{-0.008i} & 0.016e^{-1.255i} \\ 0.004e^{-0.402i} & 0.472e^{0.008i} & 0.498e^{-0.0i} & 0.015e^{-1.611i} \\ 0.009e^{0.032i} & 0.016e^{1.255i} & 0.015e^{1.611i} & 0.01e^{0.0i} \end{pmatrix}$	96.3%

Supplementary Data Table 5 | Experimental vs simulated state tomography for qubits 45

Qubits 56

	Density Matrices	Fidelity
Raw data	$\begin{pmatrix} 0.471e^{0.0i} & 0.031e^{0.392i} & 0.023e^{0.785i} & 0.442e^{-1.471i} \\ 0.031e^{-0.392i} & 0.03e^{0.0i} & 0.014e^{-2.554i} & 0.01e^{-2.85i} \\ 0.023e^{-0.785i} & 0.014e^{2.554i} & 0.024e^{0.0i} & 0.035e^{-1.983i} \\ 0.442e^{1.471i} & 0.01e^{2.85i} & 0.035e^{1.983i} & 0.475e^{0.0i} \end{pmatrix}$	91.3%
Processed data	$\begin{pmatrix} 0.481e^{0.0i} & 0.032e^{0.322i} & 0.023e^{0.723i} & 0.464e^{-1.472i} \\ 0.032e^{-0.322i} & 0.021e^{0.0i} & 0.012e^{-2.715i} & 0.012e^{-2.715i} \\ 0.023e^{-0.723i} & 0.012e^{2.715i} & 0.012e^{0.0i} & 0.038e^{-1.976i} \\ 0.464e^{1.472i} & 0.012e^{2.715i} & 0.038e^{1.976i} & 0.486e^{0.0i} \end{pmatrix}$	94.6%
Full T_2^* simulation	$\begin{pmatrix} 0.486e^{0.0i} & 0.022e^{1.851i} & 0.004e^{2.973i} & 0.49e^{-1.525i} \\ 0.022e^{-1.851i} & 0.002e^{0.0i} & 0.001e^{-1.495i} & 0.023e^{2.942i} \\ 0.004e^{-2.973i} & 0.001e^{1.495i} & 0.001e^{0.0i} & 0.004e^{1.595i} \\ 0.49e^{1.525i} & 0.023e^{-2.942i} & 0.004e^{-1.595i} & 0.51e^{0.0i} \end{pmatrix}$	98.7%
Reduced T_2^* simulation	$\begin{pmatrix} 0.483e^{0.0i} & 0.022e^{1.861i} & 0.005e^{2.481i} & 0.47e^{-1.532i} \\ 0.022e^{-1.861i} & 0.006e^{0.0i} & 0.005e^{-1.526i} & 0.022e^{3.038i} \\ 0.005e^{-2.481i} & 0.005e^{1.526i} & 0.005e^{-0.0i} & 0.004e^{1.576i} \\ 0.47e^{1.532i} & 0.022e^{-3.038i} & 0.004e^{-1.576i} & 0.506e^{0.0i} \end{pmatrix}$	96.4%

Supplementary Data Table 6 | Experimental vs simulated state tomography for qubits 56

Qubits 123

	Density Matrices	Fidelity
Raw	$\begin{pmatrix} 0.397e^{0.0i} & 0.086e^{-1.097i} & 0.063e^{0.258i} & 0.019e^{0.66i} & 0.014e^{2.445i} & 0.02e^{1.085i} & 0.031e^{-1.891i} & 0.273e^{-2.422i} \\ 0.086e^{1.097i} & 0.073e^{0.0i} & 0.029e^{1.785i} & 0.026e^{2.629i} & 0.002e^{2.976i} & 0.009e^{1.871i} & 0.011e^{-1.89i} & 0.073e^{-1.506i} \\ 0.063e^{-0.258i} & 0.029e^{-1.785i} & 0.036e^{0.0i} & 0.005e^{-0.253i} & 0.007e^{1.466i} & 0.01e^{-0.216i} & 0.007e^{0.719i} & 0.007e^{-2.843i} \\ 0.019e^{-0.66i} & 0.026e^{-2.629i} & 0.005e^{0.253i} & 0.019e^{-0.0i} & 0.003e^{-0.266i} & 0.01e^{2.866i} & 0.012e^{0.104i} & 0.025e^{1.939i} \\ 0.014e^{-2.445i} & 0.002e^{-2.976i} & 0.007e^{-1.466i} & 0.003e^{0.266i} & 0.019e^{0.0i} & 0.006e^{-1.548i} & 0.033e^{-0.018i} & 0.004e^{2.072i} \\ 0.02e^{-1.085i} & 0.009e^{-1.871i} & 0.01e^{0.216i} & 0.01e^{-2.866i} & 0.006e^{1.548i} & 0.038e^{0.0i} & 0.021e^{2.329i} & 0.051e^{-2.922i} \\ 0.031e^{1.891i} & 0.011e^{1.89i} & 0.007e^{-0.719i} & 0.012e^{-0.104i} & 0.033e^{0.018i} & 0.021e^{-2.329i} & 0.075e^{0.0i} & 0.036e^{0.225i} \\ 0.273e^{2.422i} & 0.073e^{1.506i} & 0.007e^{2.843i} & 0.025e^{-1.939i} & 0.004e^{-2.072i} & 0.051e^{2.922i} & 0.036e^{-0.225i} & 0.343e^{0.0i} \end{pmatrix}$	64.3%
Proc. data	$\begin{pmatrix} 0.446e^{-0.0i} & 0.097e^{-1.031i} & 0.073e^{0.283i} & 0.029e^{0.896i} & 0.027e^{-2.833i} & 0.023e^{1.295i} & 0.042e^{-2.219i} & 0.348e^{-2.419i} \\ 0.097e^{1.031i} & 0.023e^{-0.0i} & 0.016e^{1.478i} & 0.007e^{1.982i} & 0.009e^{-2.083i} & 0.007e^{2.616i} & 0.01e^{-1.553i} & 0.075e^{-1.427i} \\ 0.073e^{-0.283i} & 0.016e^{-1.478i} & 0.025e^{-0.0i} & 0.002e^{-0.007i} & 0.008e^{3.012i} & 0.003e^{0.796i} & 0.007e^{-2.201i} & 0.029e^{-2.789i} \\ 0.029e^{-0.896i} & 0.007e^{-1.982i} & 0.002e^{0.007i} & 0.005e^{0.0i} & 0.004e^{0.1i} & 0.002e^{-2.748i} & 0.006e^{0.066i} & 0.027e^{2.74i} \\ 0.027e^{2.833i} & 0.009e^{2.083i} & 0.008e^{-3.012i} & 0.004e^{-0.1i} & 0.017e^{-0.0i} & 0.009e^{-1.125i} & 0.02e^{0.235i} & 0.013e^{0.849i} \\ 0.023e^{-1.295i} & 0.007e^{-2.616i} & 0.003e^{-0.796i} & 0.002e^{2.748i} & 0.009e^{1.125i} & 0.046e^{0.0i} & 0.026e^{2.394i} & 0.067e^{-3.039i} \\ 0.042e^{2.219i} & 0.01e^{1.553i} & 0.007e^{2.201i} & 0.006e^{-0.066i} & 0.02e^{-0.235i} & 0.026e^{-2.394i} & 0.04e^{-0.0i} & 0.054e^{0.474i} \\ 0.348e^{2.419i} & 0.075e^{1.427i} & 0.029e^{2.789i} & 0.027e^{-2.74i} & 0.013e^{-0.849i} & 0.067e^{3.039i} & 0.054e^{-0.474i} & 0.399e^{-0.0i} \end{pmatrix}$	77.0%
Full T_2^* sim.	$\begin{pmatrix} 0.493e^{-0.0i} & 0.001e^{-1.982i} & 0.002e^{-0.351i} & 0.053e^{-2.604i} & 0.019e^{-1.276i} & 0.0e^{-0.658i} & 0.011e^{0.76i} & 0.396e^{-1.53i} \\ 0.001e^{1.982i} & 0.001e^{0.0i} & 0.0e^{0.476i} & 0.0e^{-0.572i} & 0.0e^{0.062i} & 0.0e^{-1.265i} & 0.001e^{1.531i} & 0.0e^{1.451i} \\ 0.002e^{0.351i} & 0.0e^{-0.476i} & 0.0e^{0.0i} & 0.0e^{-2.35i} & 0.0e^{-1.02i} & 0.0e^{-1.77i} & 0.0e^{1.501i} & 0.001e^{-0.814i} \\ 0.053e^{2.604i} & 0.0e^{0.572i} & 0.0e^{2.35i} & 0.017e^{0.0i} & 0.011e^{1.3i} & 0.0e^{2.095i} & 0.001e^{-2.416i} & 0.043e^{1.491i} \\ 0.019e^{1.276i} & 0.0e^{-0.062i} & 0.0e^{1.02i} & 0.011e^{-1.3i} & 0.012e^{0.0i} & 0.0e^{-1.003i} & 0.001e^{2.965i} & 0.026e^{0.633i} \\ 0.0e^{0.658i} & 0.0e^{1.265i} & 0.0e^{1.77i} & 0.0e^{-2.095i} & 0.0e^{1.003i} & 0.0e^{0.0i} & 0.0e^{-2.634i} & 0.0e^{-0.848i} \\ 0.011e^{-0.76i} & 0.001e^{-1.531i} & 0.0e^{-1.501i} & 0.001e^{2.416i} & 0.001e^{-2.965i} & 0.0e^{2.634i} & 0.002e^{0.0i} & 0.013e^{-2.335i} \\ 0.396e^{1.53i} & 0.0e^{-1.451i} & 0.001e^{0.814i} & 0.043e^{-1.491i} & 0.026e^{-0.633i} & 0.0e^{0.848i} & 0.013e^{2.335i} & 0.474e^{-0.0i} \end{pmatrix}$	87.9%
Red. T_2^* sim.	$\begin{pmatrix} 0.466e^{0.0i} & 0.003e^{-0.999i} & 0.002e^{0.256i} & 0.069e^{-2.041i} & 0.011e^{-1.024i} & 0.0e^{-0.8i} & 0.008e^{0.82i} & 0.277e^{-1.565i} \\ 0.003e^{0.999i} & 0.004e^{-0.0i} & 0.001e^{1.009i} & 0.0e^{-2.306i} & 0.0e^{-0.363i} & 0.0e^{-0.942i} & 0.002e^{1.507i} & 0.001e^{-1.168i} \\ 0.002e^{-0.256i} & 0.001e^{-1.009i} & 0.0e^{0.0i} & 0.001e^{-2.491i} & 0.001e^{-1.231i} & 0.0e^{-1.847i} & 0.0e^{1.574i} & 0.001e^{-0.912i} \\ 0.069e^{2.041i} & 0.0e^{2.306i} & 0.001e^{2.491i} & 0.041e^{0.0i} & 0.018e^{1.232i} & 0.0e^{1.362i} & 0.001e^{-2.064i} & 0.029e^{1.494i} \\ 0.011e^{1.024i} & 0.0e^{0.363i} & 0.001e^{1.231i} & 0.018e^{-1.232i} & 0.036e^{0.0i} & 0.0e^{-0.915i} & 0.002e^{-2.666i} & 0.053e^{1.266i} \\ 0.0e^{0.8i} & 0.0e^{0.942i} & 0.0e^{1.847i} & 0.0e^{-1.362i} & 0.0e^{0.915i} & 0.0e^{-0.0i} & 0.0e^{-1.978i} & 0.0e^{-0.039i} \\ 0.008e^{-0.82i} & 0.002e^{-1.507i} & 0.0e^{-1.574i} & 0.001e^{2.064i} & 0.002e^{2.666i} & 0.0e^{1.978i} & 0.004e^{0.0i} & 0.013e^{-2.489i} \\ 0.277e^{1.565i} & 0.001e^{1.168i} & 0.001e^{0.912i} & 0.029e^{-1.494i} & 0.053e^{-1.266i} & 0.0e^{0.039i} & 0.013e^{2.489i} & 0.448e^{-0.0i} \end{pmatrix}$	73.4%

Supplementary Data Table 7 | Experimental vs simulated state tomography for qubits 123

Qubits 234

	Density Matrices	Fidelity
Raw	$\begin{pmatrix} 0.455e^{0.0i} & 0.04e^{0.306i} & 0.025e^{-1.365i} & 0.007e^{-1.571i} & 0.117e^{1.214i} & 0.014e^{-2.554i} & 0.031e^{2.492i} & 0.168e^{0.295i} \\ 0.04e^{-0.306i} & 0.04e^{-0.0i} & 0.01e^{3.042i} & 0.003e^{1.249i} & 0.004e^{-0.0i} & 0.003e^{0.785i} & 0.043e^{2.489i} & 0.01e^{-0.197i} \\ 0.025e^{1.365i} & 0.01e^{-3.042i} & 0.027e^{-0.0i} & 0.007e^{2.863i} & 0.007e^{0.464i} & 0.021e^{2.253i} & 0.007e^{0.983i} & 0.021e^{1.759i} \\ 0.007e^{1.571i} & 0.003e^{-1.249i} & 0.007e^{-2.863i} & 0.033e^{0.0i} & 0.014e^{-3.142i} & 0.017e^{-1.688i} & 0.002e^{1.571i} & 0.024e^{-0.165i} \\ 0.117e^{-1.214i} & 0.004e^{0.0i} & 0.007e^{-0.464i} & 0.014e^{3.142i} & 0.07e^{0.0i} & 0.021e^{2.322i} & 0.027e^{0.993i} & 0.075e^{-2.271i} \\ 0.014e^{2.554i} & 0.003e^{-0.785i} & 0.021e^{-2.253i} & 0.017e^{1.688i} & 0.021e^{-2.322i} & 0.031e^{-0.0i} & 0.012e^{-0.427i} & 0.02e^{1.153i} \\ 0.031e^{-2.492i} & 0.043e^{-2.489i} & 0.007e^{-0.983i} & 0.002e^{-1.571i} & 0.027e^{-0.993i} & 0.012e^{0.427i} & 0.08e^{0.0i} & 0.068e^{2.843i} \\ 0.168e^{-0.295i} & 0.01e^{0.197i} & 0.021e^{-1.759i} & 0.024e^{0.165i} & 0.075e^{2.271i} & 0.02e^{-1.153i} & 0.068e^{-2.843i} & 0.265e^{0.0i} \end{pmatrix}$	52.8%
Proc. data	$\begin{pmatrix} 0.559e^{0.0i} & 0.051e^{0.236i} & 0.034e^{-1.268i} & 0.008e^{-0.245i} & 0.139e^{1.361i} & 0.021e^{-2.459i} & 0.058e^{2.693i} & 0.288e^{0.256i} \\ 0.051e^{-0.236i} & 0.011e^{-0.0i} & 0.003e^{-1.893i} & 0.001e^{-0.0i} & 0.018e^{1.18i} & 0.004e^{-2.678i} & 0.019e^{2.393i} & 0.032e^{-0.492i} \\ 0.034e^{1.268i} & 0.003e^{1.893i} & 0.003e^{0.0i} & 0.002e^{2.678i} & 0.004e^{3.142i} & 0.001e^{0.785i} & 0.005e^{-0.927i} & 0.027e^{1.916i} \\ 0.008e^{0.245i} & 0.001e^{0.0i} & 0.002e^{-2.678i} & 0.005e^{-0.0i} & 0.013e^{2.642i} & 0.004e^{-1.326i} & 0.008e^{-2.897i} & 0.028e^{-0.071i} \\ 0.139e^{-1.361i} & 0.018e^{-1.18i} & 0.004e^{-3.142i} & 0.013e^{-2.642i} & 0.068e^{-0.0i} & 0.016e^{2.313i} & 0.042e^{1.022i} & 0.089e^{-1.999i} \\ 0.021e^{2.459i} & 0.004e^{2.678i} & 0.001e^{-0.785i} & 0.004e^{1.326i} & 0.016e^{-2.313i} & 0.004e^{0.0i} & 0.012e^{-1.326i} & 0.026e^{1.532i} \\ 0.058e^{-2.693i} & 0.019e^{-2.393i} & 0.005e^{0.927i} & 0.008e^{2.897i} & 0.042e^{-1.022i} & 0.012e^{1.326i} & 0.045e^{0.0i} & 0.08e^{2.903i} \\ 0.288e^{-0.256i} & 0.032e^{0.492i} & 0.027e^{-1.916i} & 0.028e^{0.071i} & 0.089e^{1.999i} & 0.026e^{-1.532i} & 0.08e^{-2.903i} & 0.305e^{0.0i} \end{pmatrix}$	72.0%
Full T_2^* sim.	$\begin{pmatrix} 0.501e^{0.0i} & 0.002e^{-2.311i} & 0.0e^{2.872i} & 0.012e^{-2.596i} & 0.009e^{-0.058i} & 0.001e^{1.784i} & 0.011e^{0.992i} & 0.401e^{-1.621i} \\ 0.002e^{2.311i} & 0.003e^{0.0i} & 0.0e^{0.761i} & 0.0e^{-2.899i} & 0.0e^{-2.047i} & 0.0e^{-0.212i} & 0.003e^{1.792i} & 0.001e^{1.399i} \\ 0.0e^{-2.872i} & 0.0e^{-0.761i} & 0.0e^{-0.0i} & 0.0e^{-2.742i} & 0.0e^{-1.45i} & 0.0e^{-1.831i} & 0.0e^{1.549i} & 0.0e^{1.456i} \\ 0.012e^{2.596i} & 0.0e^{2.899i} & 0.0e^{2.742i} & 0.002e^{-0.0i} & 0.001e^{1.541i} & 0.0e^{-1.561i} & 0.0e^{-1.99i} & 0.009e^{1.532i} \\ 0.009e^{0.058i} & 0.0e^{2.047i} & 0.0e^{1.45i} & 0.001e^{-1.541i} & 0.002e^{-0.0i} & 0.0e^{1.91i} & 0.0e^{1.44i} & 0.005e^{-1.527i} \\ 0.001e^{-1.784i} & 0.0e^{0.212i} & 0.0e^{1.831i} & 0.0e^{1.561i} & 0.0e^{-1.91i} & 0.0e^{0.0i} & 0.0e^{1.596i} & 0.001e^{2.882i} \\ 0.011e^{-0.992i} & 0.003e^{-1.792i} & 0.0e^{-1.549i} & 0.0e^{1.99i} & 0.0e^{-1.44i} & 0.0e^{-1.596i} & 0.004e^{0.0i} & 0.013e^{-2.669i} \\ 0.401e^{1.621i} & 0.001e^{-1.399i} & 0.0e^{-1.456i} & 0.009e^{-1.532i} & 0.005e^{1.527i} & 0.001e^{-2.882i} & 0.013e^{2.669i} & 0.489e^{0.0i} \end{pmatrix}$	89.6%
Red. T_2^* sim.	$\begin{pmatrix} 0.486e^{0.0i} & 0.008e^{-1.944i} & 0.0e^{2.517i} & 0.018e^{-2.075i} & 0.007e^{0.007i} & 0.0e^{1.835i} & 0.008e^{0.797i} & 0.244e^{-1.651i} \\ 0.008e^{1.944i} & 0.017e^{-0.0i} & 0.001e^{1.218i} & 0.0e^{1.088i} & 0.0e^{2.071i} & 0.0e^{0.003i} & 0.009e^{1.745i} & 0.001e^{2.349i} \\ 0.0e^{-2.517i} & 0.001e^{-1.218i} & 0.0e^{-0.0i} & 0.0e^{3.039i} & 0.0e^{-1.004i} & 0.0e^{-1.867i} & 0.0e^{1.76i} & 0.0e^{1.609i} \\ 0.018e^{2.075i} & 0.0e^{-1.088i} & 0.0e^{-3.039i} & 0.004e^{-0.0i} & 0.002e^{1.412i} & 0.0e^{-2.537i} & 0.0e^{-2.224i} & 0.005e^{1.636i} \\ 0.007e^{-0.007i} & 0.0e^{-2.071i} & 0.0e^{1.004i} & 0.002e^{-1.412i} & 0.004e^{0.0i} & 0.0e^{-2.041i} & 0.0e^{-2.251i} & 0.005e^{1.475i} \\ 0.0e^{-1.835i} & 0.0e^{-0.003i} & 0.0e^{1.867i} & 0.0e^{2.537i} & 0.0e^{2.041i} & 0.0e^{-0.0i} & 0.0e^{-1.677i} & 0.001e^{3.008i} \\ 0.008e^{-0.797i} & 0.009e^{-1.745i} & 0.0e^{-1.76i} & 0.0e^{2.224i} & 0.0e^{2.251i} & 0.0e^{1.677i} & 0.017e^{0.0i} & 0.009e^{-3.073i} \\ 0.244e^{1.651i} & 0.001e^{-2.349i} & 0.0e^{-1.609i} & 0.005e^{-1.636i} & 0.005e^{-1.475i} & 0.001e^{-3.008i} & 0.009e^{3.073i} & 0.471e^{0.0i} \end{pmatrix}$	72.2%

Supplementary Data Table 8 | Experimental vs simulated state tomography for qubits 234

Qubits 345

	Density Matrices	Fidelity
Raw	$\begin{pmatrix} 0.419e^{-0.0i} & 0.066e^{-3.036i} & 0.021e^{1.326i} & 0.032e^{-0.351i} & 0.055e^{1.278i} & 0.014e^{2.2i} & 0.016e^{-1.695i} & 0.163e^{-1.626i} \\ 0.066e^{3.036i} & 0.045e^{-0.0i} & 0.011e^{1.107i} & 0.003e^{0.785i} & 0.006e^{2.111i} & 0.004e^{0.245i} & 0.029e^{2.971i} & 0.034e^{1.541i} \\ 0.021e^{-1.326i} & 0.011e^{-1.107i} & 0.038e^{-0.0i} & 0.009e^{-2.034i} & 0.013e^{0.322i} & 0.045e^{-0.223i} & 0.006e^{2.246i} & 0.013e^{-0.733i} \\ 0.032e^{0.351i} & 0.003e^{-0.785i} & 0.009e^{2.034i} & 0.03e^{-0.0i} & 0.029e^{0.51i} & 0.018e^{2.476i} & 0.008e^{-1.166i} & 0.009e^{-0.709i} \\ 0.055e^{-1.278i} & 0.006e^{-2.111i} & 0.013e^{-0.322i} & 0.029e^{-0.51i} & 0.053e^{-0.0i} & 0.006e^{2.111i} & 0.006e^{2.467i} & 0.063e^{-2.492i} \\ 0.014e^{-2.2i} & 0.004e^{-0.245i} & 0.045e^{0.223i} & 0.018e^{-2.476i} & 0.006e^{-2.111i} & 0.064e^{-0.0i} & 0.001e^{0.785i} & 0.02e^{0.785i} \\ 0.016e^{1.695i} & 0.029e^{-2.971i} & 0.006e^{-2.246i} & 0.008e^{1.166i} & 0.006e^{-2.467i} & 0.001e^{-0.785i} & 0.044e^{-0.0i} & 0.055e^{0.862i} \\ 0.163e^{1.626i} & 0.034e^{-1.541i} & 0.013e^{0.733i} & 0.009e^{0.709i} & 0.063e^{2.492i} & 0.02e^{-0.785i} & 0.055e^{-0.862i} & 0.308e^{-0.0i} \end{pmatrix}$	52.7%
Proc. data	$\begin{pmatrix} 0.508e^{0.0i} & 0.088e^{-3.051i} & 0.025e^{1.373i} & 0.051e^{-0.159i} & 0.084e^{1.139i} & 0.029e^{2.207i} & 0.032e^{-1.633i} & 0.295e^{-1.618i} \\ 0.088e^{3.051i} & 0.021e^{0.0i} & 0.004e^{0.983i} & 0.006e^{-2.976i} & 0.012e^{-1.92i} & 0.008e^{0.519i} & 0.008e^{2.356i} & 0.047e^{1.528i} \\ 0.025e^{-1.373i} & 0.004e^{-0.983i} & 0.023e^{-0.0i} & 0.011e^{-1.951i} & 0.006e^{0.322i} & 0.026e^{-0.19i} & 0.006e^{-2.82i} & 0.021e^{-0.82i} \\ 0.051e^{0.159i} & 0.006e^{2.976i} & 0.011e^{1.951i} & 0.009e^{0.0i} & 0.009e^{1.46i} & 0.013e^{1.966i} & 0.006e^{-1.406i} & 0.021e^{-1.279i} \\ 0.084e^{-1.139i} & 0.012e^{1.92i} & 0.006e^{-0.322i} & 0.009e^{-1.46i} & 0.023e^{-0.0i} & 0.002e^{1.107i} & 0.018e^{3.031i} & 0.085e^{-2.573i} \\ 0.029e^{-2.207i} & 0.008e^{-0.519i} & 0.026e^{0.19i} & 0.013e^{-1.966i} & 0.002e^{-1.107i} & 0.033e^{0.0i} & 0.002e^{3.142i} & 0.028e^{0.608i} \\ 0.032e^{1.633i} & 0.008e^{-2.356i} & 0.006e^{2.82i} & 0.006e^{1.406i} & 0.018e^{-3.031i} & 0.002e^{-3.142i} & 0.023e^{0.0i} & 0.067e^{0.828i} \\ 0.295e^{1.618i} & 0.047e^{-1.528i} & 0.021e^{0.82i} & 0.021e^{1.279i} & 0.085e^{2.573i} & 0.028e^{-0.608i} & 0.067e^{-0.828i} & 0.361e^{0.0i} \end{pmatrix}$	72.9%
Full T_2^* sim.	$\begin{pmatrix} 0.505e^{-0.0i} & 0.01e^{-3.135i} & 0.0e^{-1.611i} & 0.017e^{-2.681i} & 0.009e^{-0.231i} & 0.0e^{1.195i} & 0.009e^{1.029i} & 0.436e^{-1.593i} \\ 0.01e^{3.135i} & 0.001e^{-0.0i} & 0.0e^{0.719i} & 0.0e^{0.599i} & 0.0e^{3.079i} & 0.0e^{-0.815i} & 0.001e^{1.851i} & 0.009e^{1.588i} \\ 0.0e^{1.611i} & 0.0e^{-0.719i} & 0.0e^{0.0i} & 0.0e^{-2.441i} & 0.0e^{-0.707i} & 0.0e^{-1.69i} & 0.0e^{1.904i} & 0.0e^{0.198i} \\ 0.017e^{2.681i} & 0.0e^{-0.599i} & 0.0e^{2.441i} & 0.004e^{0.0i} & 0.003e^{1.874i} & 0.0e^{-1.456i} & 0.0e^{-1.86i} & 0.013e^{1.704i} \\ 0.009e^{0.231i} & 0.0e^{-3.079i} & 0.0e^{0.707i} & 0.003e^{-1.874i} & 0.003e^{-0.0i} & 0.0e^{3.088i} & 0.0e^{2.633i} & 0.004e^{-0.028i} \\ 0.0e^{-1.195i} & 0.0e^{0.815i} & 0.0e^{1.69i} & 0.0e^{1.456i} & 0.0e^{-3.088i} & 0.0e^{0.0i} & 0.0e^{-2.822i} & 0.001e^{-2.469i} \\ 0.009e^{-1.029i} & 0.001e^{-1.851i} & 0.0e^{-1.904i} & 0.0e^{1.86i} & 0.0e^{-2.633i} & 0.0e^{2.822i} & 0.001e^{0.0i} & 0.01e^{-2.652i} \\ 0.436e^{1.593i} & 0.009e^{-1.588i} & 0.0e^{-0.198i} & 0.013e^{-1.704i} & 0.004e^{0.028i} & 0.001e^{2.469i} & 0.01e^{2.652i} & 0.485e^{0.0i} \end{pmatrix}$	93.1%
Red. T_2^* sim.	$\begin{pmatrix} 0.487e^{-0.0i} & 0.011e^{-2.918i} & 0.001e^{0.452i} & 0.046e^{-1.967i} & 0.008e^{-0.127i} & 0.0e^{1.278i} & 0.006e^{0.929i} & 0.306e^{-1.619i} \\ 0.011e^{2.918i} & 0.005e^{0.0i} & 0.0e^{1.38i} & 0.001e^{1.284i} & 0.0e^{-3.015i} & 0.0e^{-0.068i} & 0.003e^{1.696i} & 0.006e^{1.584i} \\ 0.001e^{-0.452i} & 0.0e^{-1.38i} & 0.0e^{-0.0i} & 0.0e^{-2.864i} & 0.0e^{-0.839i} & 0.0e^{-1.559i} & 0.0e^{1.747i} & 0.0e^{0.092i} \\ 0.046e^{1.967i} & 0.001e^{-1.284i} & 0.0e^{2.864i} & 0.018e^{-0.0i} & 0.008e^{1.817i} & 0.0e^{-1.418i} & 0.0e^{-1.828i} & 0.01e^{1.739i} \\ 0.008e^{0.127i} & 0.0e^{3.015i} & 0.0e^{0.839i} & 0.008e^{-1.817i} & 0.017e^{-0.0i} & 0.0e^{-2.874i} & 0.001e^{-2.46i} & 0.035e^{1.259i} \\ 0.0e^{-1.278i} & 0.0e^{0.068i} & 0.0e^{1.559i} & 0.0e^{1.418i} & 0.0e^{2.874i} & 0.0e^{-0.0i} & 0.0e^{-1.608i} & 0.001e^{-2.041i} \\ 0.006e^{-0.929i} & 0.003e^{-1.696i} & 0.0e^{-1.747i} & 0.0e^{1.828i} & 0.001e^{2.46i} & 0.0e^{1.608i} & 0.004e^{0.0i} & 0.008e^{-2.818i} \\ 0.306e^{1.619i} & 0.006e^{-1.584i} & 0.0e^{-0.092i} & 0.01e^{-1.739i} & 0.035e^{-1.259i} & 0.001e^{2.041i} & 0.008e^{2.818i} & 0.468e^{0.0i} \end{pmatrix}$	78.4%

Supplementary Data Table 9 | Experimental vs simulated state tomography for qubits 345

Qubits 456

	Density Matrices	Fidelity
Raw	$\begin{pmatrix} 0.425e^{-0.0i} & 0.026e^{0.951i} & 0.038e^{2.246i} & 0.013e^{0.838i} & 0.013e^{-0.0i} & 0.008e^{-0.785i} & 0.064e^{-2.09i} & 0.298e^{-2.702i} \\ 0.026e^{-0.951i} & 0.025e^{-0.0i} & 0.015e^{0.133i} & 0.026e^{2.575i} & 0.006e^{-2.82i} & 0.004e^{0.983i} & 0.006e^{3.142i} & 0.025e^{3.062i} \\ 0.038e^{-2.246i} & 0.015e^{-0.133i} & 0.062e^{-0.0i} & 0.017e^{1.34i} & 0.013e^{-1.249i} & 0.024e^{1.785i} & 0.008e^{-1.052i} & 0.012e^{1.654i} \\ 0.013e^{-0.838i} & 0.026e^{-2.575i} & 0.017e^{-1.34i} & 0.053e^{0.0i} & 0.001e^{0.785i} & 0.005e^{1.373i} & 0.011e^{-1.661i} & 0.025e^{0.373i} \\ 0.013e^{0.0i} & 0.006e^{2.82i} & 0.013e^{1.249i} & 0.001e^{-0.785i} & 0.051e^{-0.0i} & 0.018e^{3.031i} & 0.018e^{1.107i} & 0.007e^{2.034i} \\ 0.008e^{0.785i} & 0.004e^{-0.983i} & 0.024e^{-1.785i} & 0.005e^{-1.373i} & 0.018e^{-3.031i} & 0.018e^{-0.0i} & 0.009e^{-1.46i} & 0.012e^{2.897i} \\ 0.064e^{2.09i} & 0.006e^{-3.142i} & 0.008e^{1.052i} & 0.011e^{1.661i} & 0.018e^{-1.107i} & 0.009e^{1.46i} & 0.043e^{-0.0i} & 0.043e^{0.07i} \\ 0.298e^{2.702i} & 0.025e^{-3.062i} & 0.012e^{-1.654i} & 0.025e^{-0.373i} & 0.007e^{-2.034i} & 0.012e^{-2.897i} & 0.043e^{-0.07i} & 0.323e^{-0.0i} \end{pmatrix}$	67.2%
Proc. data	$\begin{pmatrix} 0.477e^{-0.0i} & 0.012e^{1.03i} & 0.042e^{2.339i} & 0.012e^{0.54i} & 0.005e^{-1.373i} & 0.009e^{-0.464i} & 0.07e^{-2.06i} & 0.398e^{-2.7i} \\ 0.012e^{-1.03i} & 0.008e^{-0.0i} & 0.012e^{-0.245i} & 0.008e^{2.897i} & 0.001e^{-2.356i} & 0.006e^{1.249i} & 0.001e^{1.571i} & 0.026e^{3.027i} \\ 0.042e^{-2.339i} & 0.012e^{0.245i} & 0.037e^{-0.0i} & 0.008e^{3.017i} & 0.016e^{-1.695i} & 0.027e^{1.798i} & 0.006e^{0.785i} & 0.022e^{1.849i} \\ 0.012e^{-0.54i} & 0.008e^{-2.897i} & 0.008e^{-3.017i} & 0.013e^{-0.0i} & 0.004e^{-2.356i} & 0.002e^{0.0i} & 0.007e^{-2.034i} & 0.016e^{0.245i} \\ 0.005e^{1.373i} & 0.001e^{2.356i} & 0.016e^{1.695i} & 0.004e^{2.356i} & 0.014e^{-0.0i} & 0.018e^{-2.856i} & 0.007e^{0.785i} & 0.001e^{2.356i} \\ 0.009e^{0.464i} & 0.006e^{-1.249i} & 0.027e^{-1.798i} & 0.002e^{-0.0i} & 0.018e^{2.856i} & 0.027e^{-0.0i} & 0.009e^{-2.191i} & 0.012e^{-3.058i} \\ 0.07e^{2.06i} & 0.001e^{-1.571i} & 0.006e^{-0.785i} & 0.007e^{2.034i} & 0.007e^{-0.785i} & 0.009e^{2.191i} & 0.016e^{-0.0i} & 0.048e^{-0.501i} \\ 0.398e^{2.7i} & 0.026e^{-3.027i} & 0.022e^{-1.849i} & 0.016e^{-0.245i} & 0.001e^{-2.356i} & 0.012e^{3.058i} & 0.048e^{0.501i} & 0.408e^{-0.0i} \end{pmatrix}$	84.1%
Full T_2^* sim.	$\begin{pmatrix} 0.506e^{-0.0i} & 0.02e^{-3.108i} & 0.0e^{-1.711i} & 0.009e^{-2.784i} & 0.011e^{1.122i} & 0.0e^{-2.141i} & 0.011e^{-0.069i} & 0.46e^{-1.496i} \\ 0.02e^{3.108i} & 0.002e^{-0.0i} & 0.0e^{0.721i} & 0.0e^{0.354i} & 0.0e^{-2.052i} & 0.0e^{1.072i} & 0.001e^{2.121i} & 0.018e^{1.63i} \\ 0.0e^{1.711i} & 0.0e^{-0.721i} & 0.0e^{-0.0i} & 0.0e^{-1.364i} & 0.0e^{0.185i} & 0.0e^{-1.978i} & 0.0e^{1.674i} & 0.0e^{0.532i} \\ 0.009e^{2.784i} & 0.0e^{-0.354i} & 0.0e^{1.364i} & 0.001e^{0.0i} & 0.001e^{1.674i} & 0.0e^{-1.443i} & 0.0e^{3.047i} & 0.008e^{1.578i} \\ 0.011e^{-1.122i} & 0.0e^{2.052i} & 0.0e^{-0.185i} & 0.001e^{-1.674i} & 0.001e^{0.0i} & 0.0e^{-3.131i} & 0.0e^{-1.442i} & 0.011e^{-2.825i} \\ 0.0e^{2.141i} & 0.0e^{-1.072i} & 0.0e^{1.978i} & 0.0e^{1.443i} & 0.0e^{3.131i} & 0.0e^{-0.0i} & 0.0e^{0.706i} & 0.0e^{0.43i} \\ 0.011e^{0.069i} & 0.001e^{-2.121i} & 0.0e^{-1.674i} & 0.0e^{-3.047i} & 0.0e^{1.442i} & 0.0e^{-0.706i} & 0.001e^{-0.0i} & 0.012e^{-1.417i} \\ 0.46e^{1.496i} & 0.018e^{-1.63i} & 0.0e^{-0.532i} & 0.008e^{-1.578i} & 0.011e^{2.825i} & 0.0e^{-0.43i} & 0.012e^{1.417i} & 0.488e^{-0.0i} \end{pmatrix}$	95.7%
Red. T_2^* sim.	$\begin{pmatrix} 0.502e^{0.0i} & 0.019e^{-3.07i} & 0.0e^{-1.149i} & 0.014e^{-2.15i} & 0.008e^{1.123i} & 0.0e^{-2.499i} & 0.008e^{0.003i} & 0.322e^{-1.485i} \\ 0.019e^{3.07i} & 0.003e^{0.0i} & 0.0e^{1.061i} & 0.001e^{0.812i} & 0.0e^{-2.372i} & 0.0e^{1.046i} & 0.002e^{1.814i} & 0.012e^{1.648i} \\ 0.0e^{1.149i} & 0.0e^{-1.061i} & 0.0e^{-0.0i} & 0.0e^{-1.547i} & 0.0e^{-0.05i} & 0.0e^{-1.724i} & 0.0e^{1.464i} & 0.0e^{0.92i} \\ 0.014e^{2.15i} & 0.001e^{-0.812i} & 0.0e^{1.547i} & 0.005e^{-0.0i} & 0.003e^{1.592i} & 0.0e^{-1.601i} & 0.0e^{2.5i} & 0.006e^{1.544i} \\ 0.008e^{-1.123i} & 0.0e^{2.372i} & 0.0e^{0.05i} & 0.003e^{-1.592i} & 0.005e^{0.0i} & 0.0e^{-3.067i} & 0.0e^{-1.701i} & 0.011e^{2.717i} \\ 0.0e^{2.499i} & 0.0e^{-1.046i} & 0.0e^{1.724i} & 0.0e^{1.601i} & 0.0e^{3.067i} & 0.0e^{-0.0i} & 0.0e^{-0.312i} & 0.0e^{-0.553i} \\ 0.008e^{-0.003i} & 0.002e^{-1.814i} & 0.0e^{-1.464i} & 0.0e^{-2.5i} & 0.0e^{1.701i} & 0.0e^{0.312i} & 0.002e^{0.0i} & 0.011e^{-1.472i} \\ 0.322e^{1.485i} & 0.012e^{-1.648i} & 0.0e^{-0.92i} & 0.006e^{-1.544i} & 0.011e^{-2.717i} & 0.0e^{0.553i} & 0.011e^{1.472i} & 0.483e^{0.0i} \end{pmatrix}$	81.4%

Supplementary Data Table 10 | Experimental vs simulated state tomography for qubits 456

References

[1] Zajac, D. M., Hazard, T. M., Mi, X., Nielsen, E. & Petta, J. R. Scalable gate architecture for a one-dimensional array of semiconductor spin qubits. *Physical Review Applied* **6**, 054013 (2016).

[2] Baart, T. A. *et al.* Single-spin CCD. *Nature Nanotechnology* **11**, 330–334 (2016).

[3] van Diepen, C. J. *et al.* Electron cascade for distant spin readout. *Nature Communications* **12**, 1–6 (2021).

[4] Li, R. *et al.* A crossbar network for silicon quantum dot qubits. *Science advances* **4**, eaar3960 (2018).

[5] Veldhorst, M., Eenink, H. G. J., Yang, C.-H. & Dzurak, A. S. Silicon CMOS architecture for a spin-based quantum computer. *Nature Communications* **8**, 1–8 (2017).

[6] Taylor, J. M. *et al.* Fault-tolerant architecture for quantum computation using electrically controlled semiconductor spins. *Nature Physics* **1**, 177–183 (2005).

[7] Vandersypen, L. M. K. *et al.* Interfacing spin qubits in quantum dots and donors—hot, dense, and coherent. *npj Quantum Information* **3**, 1–10 (2017).

[8] Boter, J. M. *et al.* The spider-web array - a sparse spin qubit array. *arXiv:2110. 00189* (2021).

[9] Crawford, O., Cruise, J. R., Mertig, N. & Gonzalez-Zalba, M. F. Compilation and scaling strategies for a silicon quantum processor with sparse two-dimensional connectivity. *arXiv:2201. 02877* (2022).

[10] Pioro-Ladriere, M., Tokura, Y., Obata, T., Kubo, T. & Tarucha, S. Micromagnets for coherent control of spin-charge qubit in lateral quantum dots. *Applied Physics Letters* **90**, 024105 (2007).

[11] Yoneda, J. *et al.* Robust micromagnet design for fast electrical manipulations of single spins in quantum dots. *Applied Physics Express* **8**, 084401 (2015).

[12] Dumoulin Stuyck, N. I. *et al.* Low dephasing and robust micromagnet designs for silicon spin qubits. *Applied Physics Letters* **119**, 094001 (2021).

[13] Yoneda, J. *et al.* A quantum-dot spin qubit with coherence limited by charge noise and fidelity higher than 99. 9%. *Nature Nanotechnology* **13**, 102–106 (2018).

[14] Ortner, M. & Coliado Bandeira, L. G. Magpylib: A free Python package for magnetic field computation. *SoftwareX* (2020).

[15] Philips, S. uM Simulator for spin qubits (2022). URL `https://github.com/stephanlphilips/MM_sim/`.

[16] Wuetz, B. P. *et al.* Atomic fluctuations lifting the energy degeneracy in Si/SiGe quantum dots. *arXiv:2112. 09606* (2021).

[17] Noiri, A. *et al.* Radio-frequency-detected fast charge sensing in undoped silicon quantum dots. *Nano Letters* **20**, 947–952 (2020).

[18] Connors, E. J., Nelson, J. J. & Nichol, J. M. Rapid high-fidelity spin-state readout in Si/SiGe quantum dots via rf reflectometry. *Physical Review Applied* **13**, 024019 (2020).

[19] Liu, Y. Y. *et al.* Radio-frequency reflectometry in silicon-based quantum dots. *Physical Review Applied* **16**, 014057 (2021).

[20] Gühne, O. & Tóth, G. Entanglement detection. *Physics Reports* **474**, 1–75 (2009).

[21] Hradil, Z., Řeháček, J., Fiurášek, J. & Ježek, M. 3 maximum-likelihood methods in quantum mechanics. In *Quantum state estimation*, 59–112 (Springer, 2004).

[22] Watson, T. F. *et al.* A programmable two-qubit quantum processor in silicon. *Nature* **555**, 633–637 (2018).

[23] Takeda, K. *et al.* Optimized electrical control of a Si/SiGe spin qubit in the presence of an induced frequency shift. *npj Quantum Information* **4**, 1–6 (2018).
